# MEASURING IMPLEMENTATION FIDELITY OF THE TRANSITIONAL CARE MODEL IN THE MIRROR-TCM TRIAL

**DOI:** 10.1093/geroni/igad104.1878

**Published:** 2023-12-21

**Authors:** Alexandra Hanlon, Monica Ahrens, Karen Hirschman, Kathleen Mccauley, Elizabeth Shaid, Onome Osokpo, Mark Pauly, Mary Naylor

**Affiliations:** Virginia Tech, Blacksburg, Virginia, United States; Virginia Tech, Blacksburg, Virginia, United States; University of Pennsylvania, Philadelphia, Pennsylvania, United States; University of Pennsylvania, Philadelphia, Pennsylvania, United States; University of Pennsylvania, Philadelphia, Pennsylvania, United States; University of Pennsylvania, Philadelphia, Pennsylvania, United States; University of Pennsylvania, Philadelphia, Pennsylvania, United States; University of Pennsylvania, Philadelphia, Pennsylvania, United States

## Abstract

Examining the fidelity to the implementation of multi-component evidence-based interventions is essential but challenging. To capture the application of the Transitional Care Model (TCM) as designed, an advanced practice registered nurse (APRN)-led, team-based, care management strategy in a replication RCT conducted in three diverse health systems, an innovative method was utilized. This session describes the procedures, challenges, and decision-making processes associated with measuring the fidelity of the implementation of TCM’s core components over the trial. Standardized data collection forms were created for the APRNs to document the TCM elements addressed during each contact with and on behalf of the hospitalized older adults and their caregivers who had been randomly assigned to the TCM group (N=480). Measurable performance expectations for each element were established a priori (e.g., a minimum of 9 in-person visits). A dichotomous variable was created for each of the 38 elements making up the 10 TCM components which were provided monthly to each site to monitor their intervention implementation. Since not all elements were applicable to all older adults, the overall patient-level fidelity score required scaling to standardize the maximum fidelity score to 38. The individual components were inherently weighted according to their complexity as reflected by their respective number of elements. The average overall patient-level fidelity score was 34.2 (standard deviation: 3.5), median = 34.7. Higher fidelity scores indicated implementation of the minimum dose of the intervention elements. The findings provide critical insight into how to develop measures of implementation fidelity for complex multi-component evidence-based interventions.

